# *Mycobacterium heckeshornense* Infection in HIV-infected Patient

**DOI:** 10.3201/eid1611.091226

**Published:** 2010-11

**Authors:** Rabia A. Ahmed, Lil J. Miedzinski, Cary Shandro

**Affiliations:** Author affiliation: University of Alberta, Edmonton, Alberta, Canada

**Keywords:** Tuberculosis and other mycobacteria, Mycobacterium heckeshornense, bacteria, HIV, letter

**To the Editor:** *Mycobacterium heckeshornense* is a slow-growing scotochromogen phenotypically and phylogenetically related to *M. xenopi*. It was first identified as a cause of lung infection on the basis of unique 16s rRNA and 16S–23S spacer sequencing (*1*). Published data are limited to the original description and 5 case reports in English-language literature (*2**–**5*). We report disseminated *M. heckeshornense* infection in an HIV-infected patient and document its role as an emerging pathogen.

A man 40 years of age with diffuse large B-cell lymphoma had advanced HIV infection and a CD4 count <10 cells/mm^3^. Antiretroviral therapy (ART) comprising abacavir/lamivudine and lopinavir/ritonavir was initiated. Ongoing night sweats and weight loss after chemotherapy prompted submission of blood cultures, which were positive for *M. heckeshornense* after 41 days’ incubation.

Progressive wasting after 2 months’ ART prompted treatment for *M. heckeshornense* with isoniazid 300 mg 1×/d, clarithromycin 500 mg 2×/d, moxifloxacin 400 mg 1×/d, vitamin B6 25 mg 1×/d, and rifabutin 150 mg 3×/wk. Pretreatment blood and urine cultures grew *M. heckeshornense* after 41 and 30 days’ incubation, respectively. After 18 months of ART and antimycobacterial therapy, the patient’s condition improved, and his mycobacterial blood culture remained negative.

Blood for cultures was collected in Vacutainers (Becton Dickinson, Sparks, MD, USA), injected into Myco-F lytic media (Becton Dickinson), and incubated at 35–37°C in a fully automated BACTEC 9000 MB (Becton Dickinson) blood culture instrument for 42 days. Ziehl-Neelsen stain confirmed acid-fast bacilli (AFB) after 41 days. Gen-Probe AccuProbe DNA probes (Gen-Probe Incorporated, San Diego, CA, USA) for *M. avium* and *M. tuberculosis* complexes were negative. The initial blood culture was subcultured to Middlebrook 7H11 media and incubated at 35°–37°C, demonstrating a growth range of 37°–45°C (optimal growth at 42°–45°C). The isolate was forwarded to the National Reference Centre for Mycobacteriology (Winnipeg, Manitoba, Canada) for partial 16S ribosomal RNA gene sequence identification. It corresponded with 100% sequence identity to the type strain of *M. heckeshornense*. The 16S rRNA gene sequence analyzed for this isolate was 1,314 bp long and presented a divergence of 2.6% from its closest species, *M. xenopi*.

Optimal growth temperature and extended time to isolation of the initial isolate prompted a second incubation period of 42 days (total of 84 days) for all subsequent cultures. A subsequent blood culture was positive at 53 days of incubation. If negative after this reincubation period, a terminal Ziehl-Neelsen smear was performed to confirm absence of AFB.

Susceptibility testing of the initial blood and urine isolates was performed by using the radiometric broth MIC method (Becton Dickinson BACTEC 460 radiometric system; Becton Dickinson Microbiology Systems) and by microbroth dilution method (TREK Diagnostics, Cleveland, OH, USA) for isoniazid and streptomycin. Susceptibility results were as follows: amikacin MIC <2.0 μg/mL, ciprofloxacin MIC <1.0 μg/mL, clarithromycin MIC <16.0μg/mL, rifabutin MIC <0.12 μg/mL, ethambutol MIC <8.0 μg/mL, isoniazid MIC 1.0 μg/mL, and streptomycin MIC 8.0 μg/mL (*6*).

Disseminated disease caused by nontuberculous mycobacteria (NTM) has been described for HIV-infected patients with CD4 counts <50 cells/mm^3^. More than 90% of NTM cases are caused by *M. avium–intracellulare* complex (MAC) (*7*). Reports of *M. heckeshornense* infection are limited to immunocompetent persons with lung disease (*1**–**3*), tenosynovitis (*4*), and axillary lymphadenitis (*5*), attesting to the organism’s virulence.

No criteria exist for diagnosing disseminated NTM infection other than MAC, which is based on clinical signs and isolation from cultures of blood, lymph node, bone marrow, or other sterile sites. The most common diagnostic method is blood culture, positive for >90% of cases (*7*). *M. heckeshornense* was considered the etiologic agent for the patient reported here on the basis of repeated blood culture isolation, clinical signs, and improvement with treatment. Concomitant antiretroviral therapy and immune restoration also likely contributed to the patient’s improvement.

The incidence of *M. heckeshornense* infection may be underestimated because of incubation time allowed by the BACTEC 9000 series instruments at 42 days. The positive cultures for this patient were obtained close to or after the traditional 42-day period and without reincubation may have been missed. Piersimoni et al. (*8*) found that most blood cultures for *M. xenopi* were detected with terminal AFB and visual growth inspection performed after the isolates had been determined as negative by conventional means at 42 days, which suggests a need for prolonged incubation.

The optimal treatment for *M. heckeshornense* infection has not been established. Its phenotypic and genotypic resemblance to *M. xenopi* suggests that similar treatment may be reasonable. Current recommendations for treatment for *M. xenopi* infection include isoniazid, rifampin or rifabutin, and ethambutol, with or without an initial phase of streptomycin (*9*). For HIV-infected patients, consideration should be given to prolonged treatment similar to that for disseminated MAC infection, generally >12 months and accompanied by a sustained (>6 months) increase in CD4 counts to >100 cells/mm^3^ (*9*).

We report disseminated *M. heckeshornense* in an HIV-infected patient, documenting the pathogen’s increasing clinical spectrum. Its isolation requires prolonged incubation and may be missed by standard mycobacterial isolation instruments.
